# Accuracy of Four Different CT Perfusion Thresholds for Ischemic Core Volume and Location Estimation Using IntelliSpace Portal

**DOI:** 10.3390/jcdd10060239

**Published:** 2023-05-30

**Authors:** Miou S. Koopman, Jan W. Hoving, Manon L. Tolhuisen, Peng Jin, Frank O. Thiele, Linda Bremer-van der Heiden, Henk van Voorst, Olvert A. Berkhemer, Jonathan M. Coutinho, Ludo F. M. Beenen, Henk A. Marquering, Bart J. Emmer, Charles B. L. M. Majoie

**Affiliations:** 1 Department of Radiology and Nuclear Medicine, Amsterdam UMC, University of Amsterdam, 1105 AZ Amsterdam, The Netherlands; 2 Department of Biomedical Engineering and Physics, Amsterdam UMC, University of Amsterdam, 1105 AZ Amsterdam, The Netherlands; 3 Philips Medical Systems, Philips Healthcare, 5684 PC Best, The Netherlands; 4 Philips GmbH Innovative Technologies, 52074 Aachen, Germany; 5 Department of Neurology, Amsterdam UMC, University of Amsterdam, 1105 AZ Amsterdam, The Netherlands

**Keywords:** CT perfusion, DWI, stroke, endovascular thrombectomy

## Abstract

Computed tomography perfusion (CTP) is frequently used in the triage of ischemic stroke patients for endovascular thrombectomy (EVT). We aimed to quantify the volumetric and spatial agreement of the CTP ischemic core estimated with different thresholds and follow-up MRI infarct volume on diffusion-weighted imaging (DWI). Patients treated with EVT between November 2017 and September 2020 with available baseline CTP and follow-up DWI were included. Data were processed with Philips IntelliSpace Portal using four different thresholds. Follow-up infarct volume was segmented on DWI. In 55 patients, the median DWI volume was 10 mL, and median estimated CTP ischemic core volumes ranged from 10–42 mL. In patients with complete reperfusion, the intraclass correlation coefficient (ICC) showed moderate-good volumetric agreement (range 0.55–0.76). A poor agreement was found for all methods in patients with successful reperfusion (ICC range 0.36–0.45). Spatial agreement (median Dice) was low for all four methods (range 0.17–0.19). Severe core overestimation was most frequently (27%) seen in Method 3 and patients with carotid-T occlusion. Our study shows moderate–good volumetric agreement between ischemic core estimates for four different thresholds and subsequent infarct volume on DWI in EVT-treated patients with complete reperfusion. The spatial agreement was similar to other commercially available software packages.

## 1. Introduction

CT perfusion (CTP) is commonly used in the triage of acute ischemic stroke (AIS) to aid in occlusion detection, predict patient outcomes, and guide treatment decisions. CTP can provide information on cerebral hemodynamics, enabling clinicians to identify patients who may benefit from reperfusion therapies, such as intravenous thrombolysis (IVT) and endovascular treatment (EVT). The American Heart Association/American Stroke Association (AHA/ASA) and the European Stroke Organization (ESO) guidelines recommend the use of non-contrast CT and CTA to select eligible patients for IVT and EVT within early time windows [1,2]. Nevertheless, several stroke centers have integrated advanced neuroimaging with CTP into their standard imaging protocol as a helpful tool to rule out stroke mimics and identify candidates for reperfusion therapies in later time windows [3,4]. 

Over the years, many different CTP post-processing software packages have been developed that use different approaches based on cerebral blood volume (CBV), relative cerebral blood flow (rCBF), mean transit time (MTT), and time-to-maximum (Tmax) thresholds to estimate the respective ischemic core and penumbra [5,6,7,8]. Despite the widespread use of CTP, the accuracy and reliability of CTP in determining the extent of ischemic injury remain controversial, and the optimal CTP processing thresholds for accurately estimating the ischemic core and penumbra have not been established. 

IntelliSpace Portal (ISP) CT Brain Perfusion (Philips Medical Systems, Best, The Netherlands) is widely used in the routine clinical setting among other CTP software packages such as syngo.via (Siemens Healthineers, Erlangen, Germany) and RAPID (iSchemaView Inc., Menlo Park, CA, USA). While most studies focus on differences between post-processing software packages and the comparison of the CTP results between vendors, it remains largely unclear how different thresholds within the same CT perfusion software affect the accuracy of CTP core estimation relative to the infarct lesion during the follow-up [9,10,11]. Furthermore, only a few studies have investigated the spatial agreement between different CTP thresholds using syngo.via and diffusion-weighted imaging (DWI) [12,13,14], and this analysis has not been conducted with ISP. In addition, the consistency and accuracy of CTP for ischemic core estimation have been questioned. Accurate estimation of the ischemic core is critical for timely and effective stroke management. Overestimation or underestimation of the ischemic core with CT perfusion can have significant consequences, including unnecessary interventions or missed opportunities for timely treatment, which can impact patient prognosis and resource allocation. 

The aim of this study is to quantify the volumetric and spatial agreement of CTP ischemic core estimated with different thresholds using ISP and follow-up MRI infarct volume on DWI. 

## 2. Materials and Methods

### 2.1. Patient Selection

We performed a retrospective analysis of a prospectively collected registry of EVT-treated patients with baseline CTP. We included patients with AIS and large vessel occlusion who underwent EVT in our center between November 2017 and September 2020. Other inclusion criteria were admission within 24 h after symptom onset, present occlusion of the anterior circulation, successful reperfusion (defined as expanded treatment in cerebral ischemia (eTICI) score 2b–3), and approximately 24 h follow-up MRI with diffusion-weighted imaging (DWI).

### 2.2. CTP Analysis

CTP images were acquired on a dual-source 192-slice scanner (70 kVp, 12 cm coverage; SOMATOM Force, Siemens Healthcare, Forchheim, Germany) after intravenous injection of 35 mL iodinated non-ionic contrast agent (Iomeron 300, iomeprol, 300 mg iodine/mL; Bracco Imaging Deutschland GmbH, Konstanz, Germany) with an injection rate of 6 mL/s. The following protocol was used: 15 scans 1.5 s apart, followed by 15 scans 3 s apart, resulting in a total of 30 scans over a period of 60 s.

Baseline non-contrast CT and CT angiography images were acquired on the same dual-source CT scanner. Infarct volume measurements and segmentations were performed using automated post-processing analysis ISP v12.1.5 (Philips Medical Systems, Best, The Netherlands) with automated motion correction and semi-automatic brain masking. Before CTP post-processing, thin slice data were reconstructed to a slice thickness of 5 mm for noise reduction. Ischemic core was detected using four different thresholds shown in Table 1. Method 3 is the default method in ISP, and Methods 1, 2, and 4 are adjustments on the default method based on thresholds used in syngo.via (Siemens Healthcare, Erlangen, Germany) and RAPID (iSchemaView, Menlo Park, CA, USA).

### 2.3. MRI Analysis

Follow-up MRI DWI (b = 0 s/mm^2^ and b = 1000 s/mm^2^) and apparent diffusion coefficient (ADC) images were acquired on a 1.5T scanner (*n* = 43; MAGNETOM Avanto fit, Siemens Healthcare, Erlangen, Germany) or a 3.0T scanner (*n* = 14; Ingenia 3.0T, Philips Healthcare, Best, The Netherlands), depending on the availability of the scanner, both with a slice thickness of 5 mm.

Infarct volumes on DWI were segmented on DWI using a semi-automated segmentation method [15]. Visual assessment was performed by an expert neuroradiologist with >15 years of experience blinded to baseline clinical and imaging data except for the occlusion side. If necessary, the automated segmentation results were manually adjusted using ITK-SNAP [16]. We opted for DWI to evaluate follow-up infarct since it is commonly employed as a precise assessment tool for infarct core and is deemed to be more responsive to (sub)acute ischemic lesions in comparison to fluid-attenuated inversion recovery (FLAIR) [17,18]. To prevent including T2 shine-through lesions in DWI lesions, ADC maps were referenced.

### 2.4. Data Registration and Volumetric and Spatial Agreement

Elastix was used for rigid registration to align the baseline CTP with DWI [19]. Additionally, the CTP-estimated ischemic core segmentations were also co-registered to the baseline CTP using the same technique. The results were visually inspected to confirm that the images were correctly aligned, as shown in Figure S1.

### 2.5. Statistical Analysis

Frequency and summary statistics for baseline characteristics were reported. To evaluate the accuracy of the CTP software in estimating the ischemic core volume, the volume difference between the estimated CTP ischemic core and DWI follow-up infarct volume was calculated as $Volume_{difference}=Volume_{DWI}-Volume_{CTP}$. Negative values indicate overestimation of estimated ischemic core volume by CTP software.

Intraclass correlation coefficient (ICC) estimates and their 95% confidence intervals were calculated to assess the agreement between the estimated CTP ischemic core volume for each approach and the follow-up DWI infarct volume. We used a 2-way mixed-effects model with an absolute agreement and classified the degree of agreement as previously reported (ICC < 0.5 = poor agreement, ICC 0.5–0.75 = moderate agreement, ICC 0.75–0.9 = good agreement, and ICC > 0.9 = excellent agreement) [13]. Wilcoxon signed rank test and Bland-Altman analysis were conducted to compare the estimated CTP ischemic core and follow-up DWI infarct volumes. Spatial agreement between DWI and CTP core volume was assessed using the Dice similarity coefficient, sensitivity, specificity, and accuracy using EvaluateSegmentation [20]. Sensitivity was calculated as the proportion of true positive voxels on CTP (i.e., the true infarct core on DWI) divided by the total number of true positive and false negative voxels. Accuracy was calculated as the proportion of true positive (infarct) and true negative (no infarct) on CTP divided by the total number of CTP voxels. Last, a sensitivity analysis was conducted to compare patients who achieved complete reperfusion with those who achieved successful but incomplete reperfusion (i.e., eTICI 3 vs. eTICI 2b–2c). All statistical analyses were performed using R (R Core Team (2020). R: A language and environment for statistical computing, R Foundation for Statistical Computing, Vienna, Austria. https://www.R-project.org, accessed on 24 May 2023).

## 3. Results

Two hundred eighty-four patients were presented to our comprehensive stroke center for EVT between November 2017 and October 2020 and received baseline CTP imaging. Eighty-four (30%) patients had follow-up MRI after CTP with a median of 23 h (IQR 18–34). After the application of the exclusion criteria, we included a total of 55 patients in our analysis (Figure S2). Out of the 55 patients who were included, 49 were part of either MR CLEAN-NO IV, MR CLEAN-MED, or MR CLEAN-LATE trials from the CONTRAST consortium [21]. These patients underwent follow-up imaging according to the pre-defined imaging protocol of the respective trial. Patient characteristics and outcomes are shown in Table 2 and Table 3. The median age was 71 (IQR 58–77) years, the median NIHSS was 15, and most patients presented within 6 h of symptom onset (52/55).

### 3.1. Volumetric Comparison CTP and MRI

**Figure 1:**
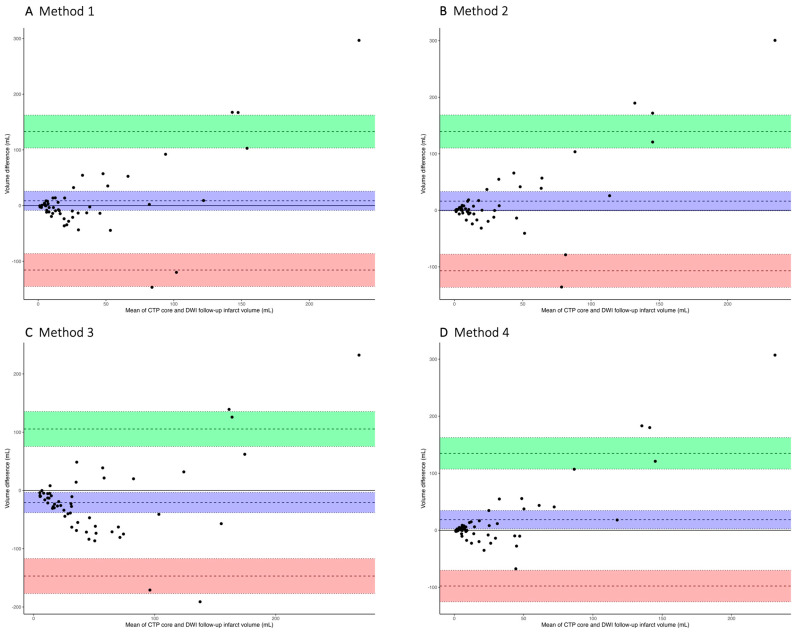
Bland-Altman plots comparing the CTP ischemic core volume and DWI follow-up infarct volume for Method 1 (**A**), Method 2 (**B**), Method 3 (**C**), and Method 4 (**D**). The mean bias (blue), **lower** (red), and **upper** (green) limits of agreements with 95% CIs are shown. Values closer to 0 indicate better agreement between CTP ischemic core and follow-up DWI infarct volume; negative values indicate overestimation by CTP. CTP = computed tomography perfusion; DWI = diffusion-weighted imaging.

Median follow-up DWI infarct volume was 10 mL. Median ischemic core volumes were 19 mL, 11 mL, 42 mL, and 10 mL estimated with Methods 1, 2, 3, and 4, respectively. Median volumetric differences between follow-up DWI infarct and CTP ischemic core were −2 (−19), 1 (−5–17), −24 (−56–−5), and 2 (−4–14) for all methods (Table 4). ICC estimates show poor volumetric agreement for all methods (Method 1 ICC 0.40 (95% CI 0.14–0.59), Method 2 ICC 0.36 (95% CI 0.11−0.57), Method 3 ICC 0.45 (95% CI 0.21–0.64), and Method 4 ICC 0.38 (95% CI 0.14–0.58). Bland-Altman plots for volumetric agreement between CTP core and DWI infarct volume are shown in Figure 1. A negative bias in Figure 1 indicates ischemic core overestimation by CTP.

### 3.2. CTP Core Overestimation

**Figure 2:**
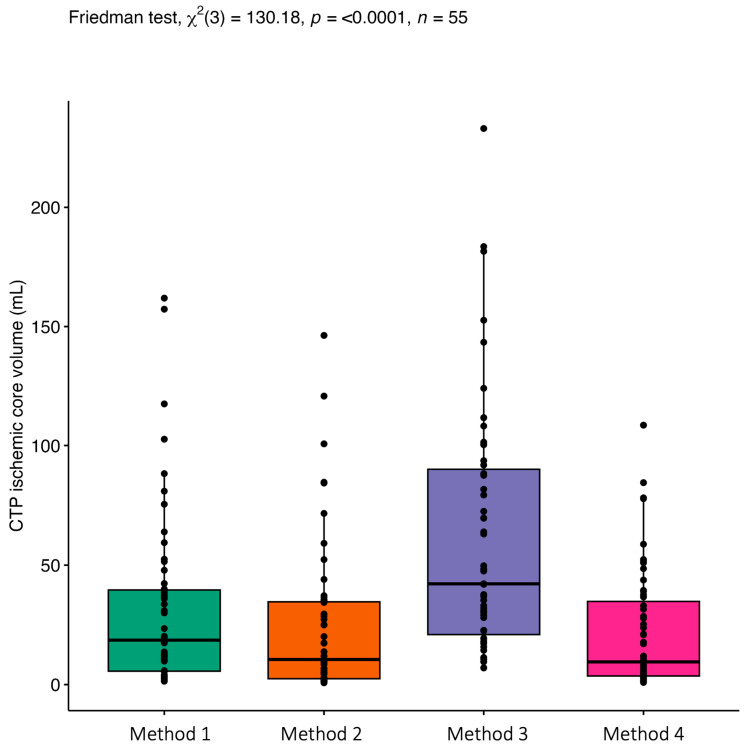
Boxplots of estimated CTP ischemic core distribution for all four methods. Boxplots represent the median and interquartile range. Friedman test showed significant differences between core estimation methods, χ^2^ = 130.18, *p* = <0.001. Pairwise Wilcoxon signed rank test comparison shows significant differences in ischemic core volume between all methods (*p* < 0.001) except for Method 2-Method 4 (*p* = 0.8).

Scatter plots of CTP core and DWI volumes divided by occlusion location are shown in Figure S3. More than 10 mL overestimation of CTP core was seen in 17/55 (31%), 10/55 (18%), 36/55 (65%), and 10/55 (18%) patients with Methods 1, 2, 3, and 4, respectively. Severe overestimation of >50 mL was most common with Method 3 (15/55; 27%). In Methods 1 (2/55; 4%), 2 (2/55;4%), and 4 (1/55;2%), severe overestimation was only seen in patients with carotid-T occlusion. The Boxplot of the estimated CTP core distribution is shown in Figure 2. In all methods, overestimation was more frequent in patients with carotid-T occlusion (Figure S4).

### 3.3. Spatial Agreement

The median Dice similarity coefficient was 0.17 (0.03–0.31), 0.17 (0.02–0.33), 0.19 (0.04–0.30), and 0.19 (0.03–0.31) for Methods 1–4, respectively. Table 4 shows the spatial overlap between the ischemic core on CTP and the final infarct on MRI.

### 3.4. Incomplete versus Complete Reperfusion

Results of the sensitivity analysis for patients with complete reperfusion (eTICI 3; *n* = 29) versus incomplete (eTICI 2b–2c; *n* = 26) are shown in Table 5. Median DWI infarct volume in patients with complete reperfusion was 18 mL, and estimated ischemic core volumes were 13 mL, 10 mL, 37 mL, and 9 mL for Methods 1–4, respectively. In patients with incomplete reperfusion, the median infarct volume was 9 mL, and estimated ischemic core volumes were 20 mL, 14 mL, 50 mL, and 10 mL. ICC estimates were good (0.76 (0.55–0.88) for Method 1; they were moderate 0.69 (0.45–0.84), 0.56 (0.01–0.81), and 0.71 (0.48–0.85) for Methods 2, 3, and 4 in patients with complete reperfusion. ICCs were poor in the subgroup with incomplete reperfusion status for all methods.

## 4. Discussion

Our study shows moderate—good volumetric agreement between ischemic core estimates for four different thresholds and subsequent infarct volume on DWI in EVT-treated patients with complete reperfusion (eTICI 3). Poor volumetric agreement was found in all successfully reperfused patients (eTICI 2b–3), which could be caused by a delay between imaging and reperfusion or infarct growth after endovascular treatment. The median DWI infarct volume was consistently lower compared with the CTP ischemic core volume estimated with various methods, indicating that all methods tend to overestimate the extent of the ischemic core.

The overall spatial agreement was low with a Dice range between 0.17 and 0.19. This is comparable to other commercially available software packages described previously [11,12,22]. In a study using RAPID, pretreatment ischemic core volume in patients who had more than 50% angiographic reperfusion was compared with follow-up MRI at 24 h. They found a median Dice similarity coefficient of 0.24 (IQR 0.15–0.37) [11]. There are several reasons why the Dice similarity coefficient between CTP core volume and DWI infarct volume may be overall low. CTP is performed within the first few hours after stroke onset, while DWI in our study was performed at a later stage (after approximately 24 h). This temporal difference can result in changes in the size and shape of the infarct, leading to a poorer overlap between the two measures. Additionally, spatial resolution differences between CTP and MRI can affect the accuracy of measurements. Therefore, the Dice score to select the best-performing method should always be used with caution, especially in patients with very small infarct volumes or a scattered infarct pattern.

The accuracy of CTP in estimating the ischemic core and penumbra has been extensively studied in the literature, and it is well established that different post-processing software packages can produce varying results due to differences in their algorithms, processing techniques, and post-processing tools [10]. Austein et al. investigated the volumetric performance of three commercially available software packages—Olea Sphere, syngo.via, and RAPID—to predict the final infarct volume after thrombectomy in patients with AIS. They found that all three software packages showed a high correlation with the final infarct volume, but RAPID demonstrated the strongest correlation with the final infarct volume (r = 0.93), followed by Olea Sphere (r = 0.87), and syngo.via (r = 0.84) [9]. A study by Psychogios et al. compared four software packages (RAPID, VEOcore, syngo.via, and Olea) that were used for the automated perfusion calculations. The study found significant differences in the core volumes estimated by the different software packages, with volume differences of up to 33 mL [23].

Although several studies have investigated the optimal CTP processing thresholds for accurately estimating the ischemic core and penumbra, there is currently no consensus on the best approach. Some studies have suggested that using CBV or Tmax thresholds can accurately identify the ischemic core, while others have found that rCBF or MTT thresholds may be more accurate. ISP relies on CBV thresholds for the estimation of ischemic core, which has been also used in other software packages [10,24,25].

Accurate estimation of the ischemic core volume is beneficial for optimal stroke management, particularly when using CTP to select patients for reperfusion therapy [1,2,4,26]. Intravenous thrombolysis has been proven safe and effective in patients with salvageable brain tissue who present outside the standard 4.5 h time window or with an unknown time of onset [4]. Recent large core trials (SELECT-2 and ANGEL-ASPECTS) have shown the efficacy of EVT up to 24 h from stroke onset in patients with LVO and large cores [27,28]. Therefore, despite being a negative prognostic factor for the functional outcome, the presence of a large core volume alone should not be an absolute contraindication for EVT. Careful interpretation of CT perfusion results and clinical correlation is essential to ensure appropriate and timely management of ischemic stroke patients. Underestimation of the ischemic core can be caused by several factors, including infarct growth between baseline CTP and follow-up MRI, and can increase the risk of intracranial hemorrhage due to IVT or EVT [29]. The consequences of overestimation are much higher, especially when CTP may deem patients ineligible for reperfusion therapy, even if they could potentially benefit. Overestimation of the ischemic core (‘ghost core concept’) has been described especially in hyperacute settings and refers to an area of decreased perfusion that is seen on CTP but is not actually present [30,31]. The etiology of the ghost core phenomenon remains unclear, although it is hypothesized to be influenced by the timing of imaging acquisition. Notably, it is important to recognize that CTP evaluates the hemodynamic status of the brain at a certain time point and does not reflect actual tissue viability [29]. Moreover, shorter periods of severe hypoperfusion are less likely to result in permanent injury.

The results of our study showed that overestimation of the CTP core volume by more than 10 mL occurred in a significant proportion of patients, ranging from 18% to 65% depending on the core estimation approach used. It is unlikely that an overestimation of the ischemic core of 10 mL would have a substantial effect on the treatment decision for endovascular therapy. However, severe overestimation of more than 50 mL might falsely withhold patients from endovascular or intravenous treatment. Severe overestimation was most common with Method 3 (CBV < 2.0 mL/100 mg), occurring in 27% of patients. Severe overestimation was infrequently seen in Methods 1, 2, and 4, ranging from 2–4%. However, it is worth noting that severe overestimation was only seen in patients with carotid-T occlusion in these methods. This suggests that occlusion location may have an impact on the accuracy of CTP processing and ischemic core estimation.

This study has several strengths, including the utilization of a single acquisition protocol for CTP, which minimized the variability in the source data allowed for consistent comparison. Additionally, the assessment of follow-up infarct volume using DWI at a median time of approximately 24 h after reperfusion is a notable strength, as it helps to reduce the impact of brain edema-induced infarct volume inflation on the results. Moreover, DWI is considered the “gold standard” for assessing the extent of infarction in ischemic stroke [18].

There are several limitations to our study that should be noted. First, our sample size was relatively small, which may affect the generalizability of our findings. Second, our study may be susceptible to selection bias because follow-up MRI is not a standard practice in the Netherlands. The majority of patients in our dataset were enrolled in one of the following trials: MR CLEAN-NO IV (ISRCTN80619088), MR CLEAN-MED (ISRCTN76741621), or MR CLEAN-LATE (ISRCTN19922220)—all of which are part of the CONTRAST consortium [21]. According to the standard follow-up imaging protocol of each respective trial, these patients underwent follow-up imaging. It should be noted that DWI has its own limitations, such as potential false positives due to T2 shine-through effects or false negatives in the hyperacute phase of stroke. However, by using ADC maps, we mitigated the likelihood of incorporating T2 shine-through lesions. Third, most of the patients in our study (42/55) were scanned during the hyperacute window (<90 min of onset), with a median time from onset to imaging of 85 min. Overestimation has been reported in this time frame, and as such, patient CTP selection should not be recommended during this early window [31]. Fourth, this study was conducted at a single center which may limit the generalizability of the findings to other settings or populations. External validation may be needed to confirm the applicability of the results in different clinical settings. Fifth, it should be noted that we did not assess the accuracy of penumbra estimation in our study, as only patients who underwent successful reperfusion were included. Furthermore, the accuracy of the mismatch ratio, which is an important parameter for making decisions in stroke management, was not evaluated. Last, as we have previously described, co-registration errors between CT and MRI may have contributed to lower Dice scores. Additionally, the reliability of CTP can be affected by various factors. For example, movement during image acquisition can lead to image artifacts and reduced image quality, which can impact the accuracy of CTP-derived measurements. Furthermore, changes in a patient’s cardiac output or blood pressure can affect the contrast delivery to the brain, which can impact the accuracy of CTP measurements. Therefore, it is important to interpret CTP images in conjunction with other imaging modalities and clinical information to improve the accuracy of diagnosis and treatment decisions.

## 5. Conclusions

In patients with complete reperfusion after EVT, a volumetric agreement between CTP and MRI was moderate-good. In successfully reperfused patients, Philips IntelliSpace Portal CTP ischemic core estimation showed spatial agreements similar to other commercially available software packages. Overestimation of the ischemic core was most common in Method 3 and was observed with a greater frequency in patients displaying carotid-T occlusion.

## Figures and Tables

**Table 1:** The threshold for estimation of ischemic core volumes.

	Ischemic Core
Method 1	CBV < 1.2 mL/100 g and rMTT > 150%
Method 2	CBV < 1.3 mL/100 g and rMTT > 250%
Method 3	CBV < 2.0 mL/100 g and rMTT > 150%
Method 4	CBV < 1.3 mL/100 g and rTmax > 6 s

CBV, Cerebral Blood Volume; MTT, Mean Transit Time; Tmax, Time to Maximum. Relative MTT (rMTT) is the ratio between the MTT on both hemispheres. Relative Tmax (rTmax) is the subtraction between the Tmax maps on both hemispheres.

**Table 2:** Baseline characteristics.

	Sample Size (*n* = 55)
**Patient Characteristics**	
Age (year)—median (IQR)	71 (58–77)
Female (%)	21 (38)
Baseline NIHSS—median (IQR)	15 (10–18)
Time from onset to reperfusion (min)—median (IQR)	201 (137–296)
Time from onset to imaging—median (IQR)	85 (58–189)
**Imaging Characteristics**	
Occlusion location on CTA-*n* (%)	
Intracranial ICA	3 (5)
ICA-T	8 (15)
M1	40 (73)
M2	4 (7)
ASPECTS—median (IQR)	8 (7–10)
Ischemic core volume on CTP (mL)—median (IQR)	
Method 1	19 (6–40)
Method 2	11 (2–35)
Method 3	42 (21–90)
Method 4	10 (4–38)

ASPECTS; Alberta Stroke Program Early CT Score; ICA, internal carotid artery; ICA-T, internal carotid artery terminus; IQR, interquartile range; NIHSS, National Institute of Health Stroke Scale; CTA, CT angiography, CTP, CT perfusion; IVT, intravenous alteplase.

**Table 3:** Outcomes of EVT-treated patients.

	Sample Size (*n* = 55)
Characteristics	
Functional independence (mRS 0–2) at 90 days-*n* (%)	30 (77)
eTICI score-*n* (%)	
2b	19 (34)
2c	7 (13)
3	29 (53)
DWI volume (mL)—median (IQR)	10 (5–38)
Time between CTP and MRI (hrs)—median (IQR)	23 (18–34)

IQR, interquartile range; CTP, CT perfusion; DWI, diffusion-weighted imaging; eTICI, expanded treatment in cerebral ischemia.

**Table 4:** Volumetric and spatial agreement between CTP ischemic core and DWI infarct.

	Volume Difference in mL—Median (IQR)	Dice—Median (IQR)	Sensitivity—Median (IQR)	Specificity—Median (IQR)
Method 1	−2 (−13–9)	0.17 (0.03–0.31)	0.22 (0.06–0.36)	1.00 (0.99–1.00)
Method 2	1 (−5–17)	0.17 (0.02–0.33)	0.16 (0.05–0.37)	1.00 (1.00–1.00)
Method 3	−24 (−56–−5)	0.19 (0.04–0.30)	0.41 (0.21–0.60)	0.99 (0.99–1.00)
Method 4	2 (−4–14)	0.19 (0.03–0.31)	0.16 (0.02–0.36)	1.00 (1.00–1.00)

IQR, interquartile range.

**Table 5:** Volumetric agreement for patients with incomplete (eTICI 2b-c) versus complete (eTICI 3) endovascular reperfusion.

	Method 1	Method 2	Method 3	Method 4
**Incomplete reperfusion (*n* = 26)**				
Volume difference in mL—median (IQR)	2 (−11–35)	3 (0–41)	−10 (−30–21)	6 (−1–37)
ICC (95% CI)	0.26 (−0.12–0.58)	0.23 (−0.15–0.56)	0.40 (0.03–0.68)	0.24 (−0.14–0.57)
Dice—median (IQR)	0.15 (0.02–0.32)	0.13 (0.01–0.33)	0.23 (0.04–0.32)	0.12 (0.02–0.33)
**Complete reperfusion (*n* = 29)**				
Volume difference in mL—median (IQR)	−3 (−14–2)	0 (−6–5)	−34 (−61–−13)	0 (−8–6)
ICC (95% CI)	0.76 (0.55−0.88)	0.69 (0.45−0.84)	0.56 (0.01–0.81)	0.71 (0.48–0.85)
Dice—median (IQR)	0.20 (0.09–0.31)	0.20 (0.08–0.32)	0.19 (0.04–0.31)	0.23 (0.04–0.31)

ICC, intraclass correlation coefficient; IQR, interquartile range; eTICI, expanded treatment in cerebral ischemia.

## Data Availability

Individual patient data cannot be made available under Dutch law as we did not obtain patients’ approval for sharing individual coded patient data. In line with privacy regulations, the publication of individual patient data as well as syntax files and outputs of statistical analyses is forbidden by the Data Privacy Officer of the Amsterdam UMC. All syntax files and outputs of statistical analyses are available upon reasonable request made to the corresponding author.

## Supplementary Materials

The following supporting information can be downloaded at: https://www.mdpi.com/article/10.3390/jcdd10060239/s1, Figure S1: Example of co-registration of segmented ischemic core volume op CTP (red) and follow-up infarct volume on DWI (blue) projected on smoothened CTP CBV map (grey) in a case with moderate volumetric and spatial accuracy; Figure S2: Flowchart of patient selection; Figure S3: Scatter plots show the agreement between the estimated CTP ischemic core volume and the follow-up DWI infarct volume for (A) method 1, (B) method 2, (C) method 3 and method 4 (D). The dotted line represents the identity line. Points below this line indicate a larger CTP ischemic core volume compared to the follow-up DWI lesion, i.e., overestimation by CTP. Points above the identity indicate underestimation by CTP or infarct growth between baseline and follow up imaging. CTP = CT perfusion; DWI = diffusion weighted imaging; Figure S4: Boxplots of ischemic core overestimation per occlusion location. Boxplots represent median and interquartile range. Negative values represent overestimation of ischemic core.
