# Supplementary material for: Accuracy of Four Different CT Perfusion Thresholds for Ischemic Core Volume and Location Estimation Using IntelliSpace Portal
Source: J Cardiovasc Dev Dis. 2023 May 30;10(6):239. doi: 10.3390/jcdd10060239 (PMC10299344; doi:10.3390/jcdd10060239)
Supplement: Supplementary file 1 [file jcdd-10-00239-s001.zip › jcdd-2367982-supplementary.pdf]

SUPPLEMENTARY MATERIAL

Figure S1. Example of co-registration of segmented ischemic core volume op CTP (red) and follow-up infarct volume on DWI (blue) projected on smoothened CTP CBV map (grey) in a case with moderate volumetric and spatial accuracy.

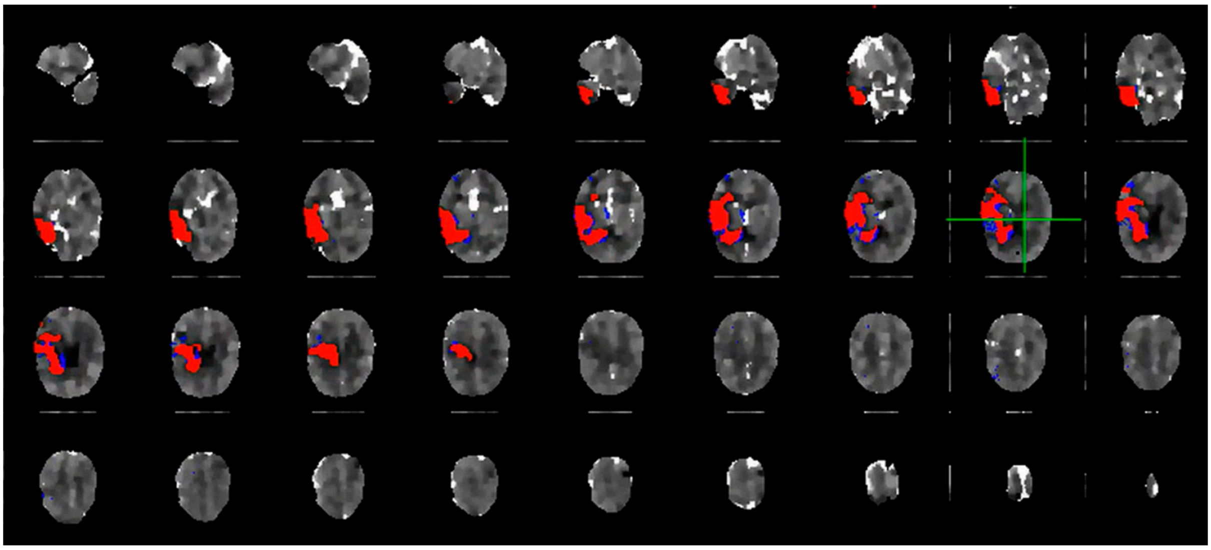

Figure S2. Flowchart of patient selection.

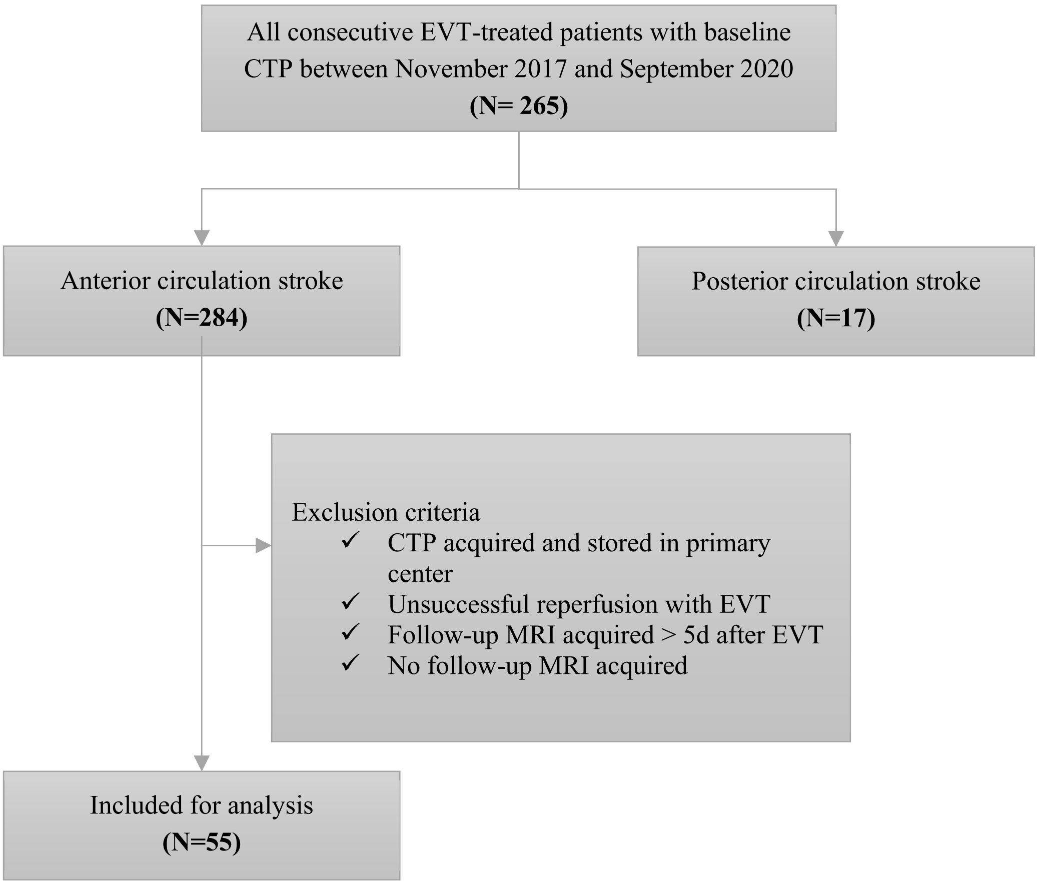

Figure S3. Scatter plots show the agreement between the estimated CTP ischemic core volume and the follow-up DWI infarct volume for (A) method 1, (B) method 2, (C) method 3 and method 4 (D). The dotted line represents the identity line. Points below this line indicate a larger CTP ischemic core volume compared to the follow-up DWI lesion, i.e. overestimation by CTP. Points above the identity indicate underestimation by CTP or infarct growth between baseline and follow up imaging. CTP = CT perfusion; DWI = diffusion weighted imaging

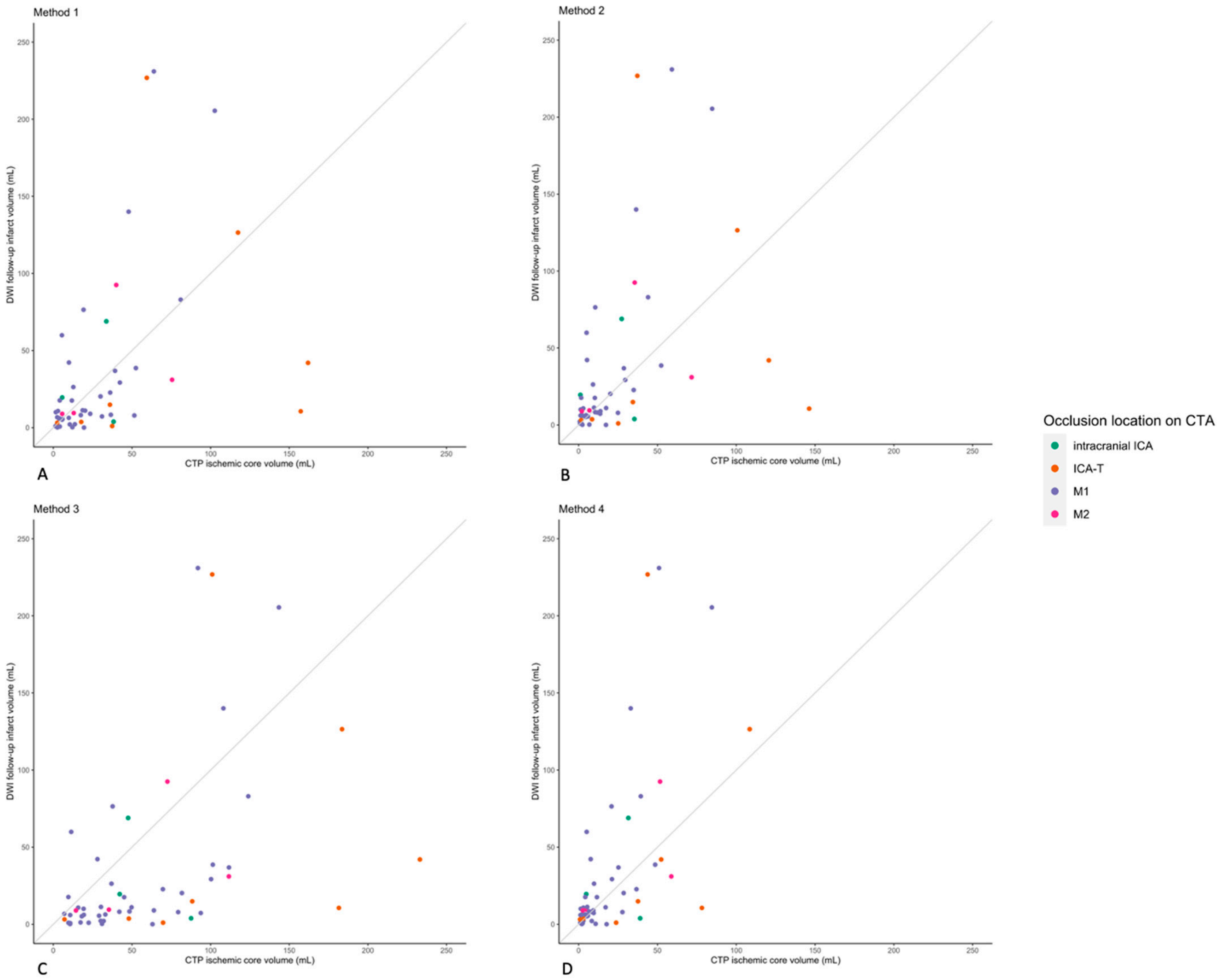

Figure S4. Boxplots of ischemic core overestimation per occlusion location. Boxplots represent median and interquartile range. Negative values represent overestimation of ischemic core.

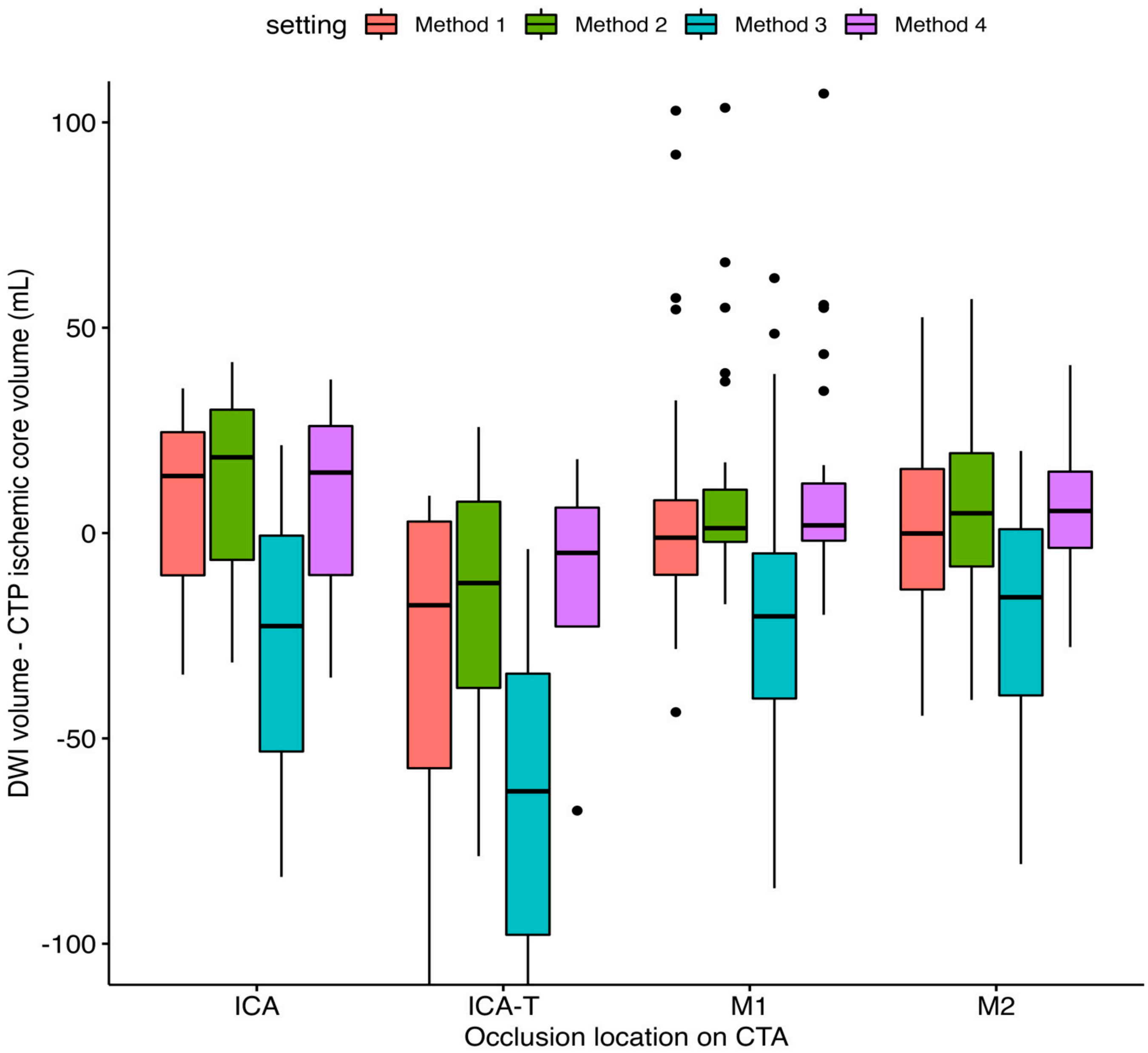
setting
Method 1
Method 2
Method 3
Method 4
100
50
0
-50
-100
DWI volume - CTP ischemic core volume (mL)
ICA
ICA-T
M1
M2
Occlusion location on CTA
